# Pre-Eclampsia and Eclampsia: An Update on the Pharmacological Treatment Applied in Portugal †

**DOI:** 10.3390/jcdd5010003

**Published:** 2018-01-17

**Authors:** Gonçalo Miguel Peres, Melissa Mariana, Elisa Cairrão

**Affiliations:** CICS-UBI, Health Sciences Research Centre, University of Beira Interior, Av. Infante D. Henrique, 6200-506 Covilhã, Portugal; goncalomperes@gmail.com (G.M.P.); melissa.r.mariana@gmail.com (M.M.)

**Keywords:** pre-eclampsia, eclampsia, pharmacological therapy, pathophysiology, Portugal

## Abstract

Pre-eclampsia and eclampsia are two hypertensive disorders of pregnancy, considered major causes of maternal and perinatal death worldwide. Pre-eclampsia is a multisystemic disease characterized by the development of hypertension after 20 weeks of gestation, with the presence of proteinuria or, in its absence, of signs or symptoms indicative of target organ injury. Eclampsia represents the consequence of brain injuries caused by pre-eclampsia. The correct diagnosis and classification of the disease are essential, since the therapies for the mild and severe forms of pre-eclampsia are different. Thus, this review aims to describe the most advisable antepartum pharmacotherapy for pre-eclampsia and eclampsia applied in Portugal and based on several national and international available guidelines. Slow-release nifedipine is the most recommended drug for mild pre-eclampsia, and labetalol is the drug of choice for the severe form of the disease. Magnesium sulfate is used to prevent seizures caused by eclampsia. Corticosteroids are used for fetal lung maturation. Overall, the pharmacological prevention of these diseases is limited to low-dose aspirin, so it is important to establish the safest and most effective available treatment.

## 1. Introduction

Pregnancy is characterized by significant metabolic and hemodynamic changes that begin early in the gestational period. Major hemodynamic changes include an increase in the cardiac output during the first trimester, sodium and water retention leading to plasma volume expansion with a peak around week 30, and reductions in the systemic vascular resistance and systemic blood pressure [1]. The reduction of the systemic vascular resistance is around 25% and is due to the increase in vasodilating agents, like nitric oxide and prostacyclin production, and the decrease in the sensitivity to norepinephrine and angiotensin [1]. The diastolic blood pressure begins to decrease from the 7th week of gestation, with a 10 mmHg decline between the 24th–26th gestation weeks, returning to normal values during the third trimester [2,3]. These are some of the changes that can occur during pregnancy. Hypertension is the most prevalent maternal complication worldwide (several studies estimate that it affects 7–10% of all pregnancies) [4,5], and it is associated with a significant morbidity and mortality of the mother and fetus. In fact, hypertension is the second largest cause of direct maternal death worldwide (14% of the total) [6], and it is estimated that 192 people die every day because of hypertensive disorders in pregnancy [7]. Pre-eclampsia and eclampsia are two hypertensive disorders of pregnancy, considered as major causes of maternal and perinatal morbidity and mortality [5]. These diseases affect between 3% and 5% of all pregnancies and account for more than 60,000 maternal and 500,000 fetal deaths per year worldwide [8]. It is known that pre-eclampsia and eclampsia are the hypertensive disorders that involve the most significant health risks for the pregnant woman and the fetus. In this context, it is imperative to evaluate whether all possible and necessary measures are being taken correctly in terms of prevention, maintenance, and treatment of the disease. Gathering pharmacological information from Portuguese and International guidelines, the main purpose of this review is to describe the most recommended pharmacological treatments for these two hypertensive disorders in pregnant women during the gestational and antepartum period.

## 2. Methods

A literature review was performed based on the analysis of guidelines and papers available on PubMed. This search was carried out for pre-eclampsia, eclampsia, and for the pharmacological therapy, using different combinations of several keywords, such as pre-eclampsia, eclampsia, pharmacology, therapy, pregnancy diseases, pathophysiology, cardiovascular diseases (CVD), pregnancy, and hypertensive disorders of pregnancy, only present in the title, the abstract, or both. The search terms used were pre-eclampsia OR eclampsia AND pharmacology; pre-eclampsia OR eclampsia AND pathophysiology; pre-eclampsia OR eclampsia AND therapy; pregnancy diseases AND pre-eclampsia OR eclampsia; CVD AND pregnancy; hypertensive disorders of pregnancy AND pre-eclampsia OR eclampsia. From all the articles retrieved, unrelated, inaccessible, duplicate, and foreign language papers were excluded. The bibliographies of the articles used in this review were searched for additional relevant citations. The search was emphasized for the last six years (2011–2017), however, the results of the most important studies and those with greater relevance for this review are described below, and a weight-of-evidence approach was applied. In addition to PubMed, several documents and guidelines available from different national and international hospitals and organizations were also analyzed.

## 3. Pre-Eclampsia and Eclampsia

Pre-eclampsia is a multisystemic disease characterized by the development of hypertension after 20 weeks of gestation in a previously normotensive woman, with the presence of proteinuria or, in its absence, of signs or symptoms indicative of target organ injury [9]. The clinical signs involve multiple organs, including the liver, kidneys, heart, lungs, brain, and pancreas (Table 1). These complications can result in maternal and fetal adverse outcomes that can lead to intrauterine growth restriction, placental hypoperfusion, premature placental disruption or, in most serious situations, termination of pregnancy and fetal and maternal death [10,11].

This disease can be divided into mild and severe forms, according to the severity and type of the symptoms presented. The mild form of pre-eclampsia is characterized by systolic blood pressure (SBP) ≥140 mmHg or diastolic blood pressure (DBP) ≥90 mmHg, and proteinuria >300 mg/24 h [12,13]. The severe form of pre-eclampsia is characterized by severe hypertension (SBP > 160 mmHg or DBP > 110 mmHg), or severe proteinuria (>2 g/24 h), or signs and symptoms of target organ damage [12,13]. Women with severe pre-eclampsia may present headaches, visual disturbances (including blindness), epigastric pain, nausea and vomits, hepatic and renal insufficiency, and pulmonary edema [14].

The incidence of pre-eclampsia is also explained by several risk factors (described in Table 2), that include maternal age under 20 years old or over 40 years old, history of pre-eclampsia, previous hypertension, autoimmune diseases, and obesity [15,16]. A woman is at moderate risk for pre-eclampsia if she has no more than one risk factor (Table 2); a woman is at high risk for pre-eclampsia if she has two or more risk factors for the disease [12,16]. According to this classification, the clinician will consider the prescription of low-dose aspirin to the patient (this will be discussed further in the results).

On another strand, a surprising discovery was made consisting in the demonstration that smoking protects pregnant women from developing pre-eclampsia [17], since smoking enhances the expression of ligands of the vascular endothelial growth factor (VEGF) family, which regulate the differentiation and survival of cytotrophoblasts, leading to normal uterine invasion [18]. Nonetheless, it is still not recommended that pregnant women smoke, since smoking is a risk factor for several complications during pregnancy, namely miscarriages, placental abruption, preterm delivery, and reduced birth weight [18].

Eclampsia represents the consequence of brain injuries caused by pre-eclampsia. It is defined as pre-eclampsia with the abrupt development of seizures or coma during the gestational period or post-partum, non-attributable to other neurologic diseases that can justify the convulsive state (namely epilepsy or cerebral stroke) [9]. Eclampsia is the rarest [23] and most severe [24] of all the hypertensive disorders of pregnancy, with a high maternal and fetal mortality [25].

Pre-eclampsia is associated with several complications not only during pregnancy but also in postpartum period. A broad diversity of studies has demonstrated that women who had pregnancies complicated with pre-eclampsia have, throughout live, a greater risk and incidence of cardiovascular diseases, with an adjusted hazard ratio of 2.1 in a 95% confidence interval of 1.8–2.4 according to Ray and collaborators [26,27,28], major cardiovascular events, such as myocardial infarction (with an adjusted hazard ratio of 13.0 in a 95% confidence interval of 4.6–6.3), stroke (with an adjusted hazard ratio of 14.5 in a 95% confidence interval of 1.3–165.1), or heart failure (with an adjusted hazard ratio of 8.3 in a 95% confidence interval of 4.2–16.4) [29], and hospitalization related with cardiovascular events [30]. Children born from women who had pre-eclampsia during their pregnancies are also at a greater risk for cardiovascular events during their lifetime [31]. Other studies demonstrated an elevated blood pressure and body mass index in these children [32]. Therefore, pregnancy can be considered as a window for the future health of women and their children.

It is known that, currently, the only definitive cure for pre-eclampsia is the delivery of the fetus, and available therapies for this disease only have symptom management purposes [5]. For these reasons, it is of major importance that the pharmacological prophylaxis treatment is as effective and safe as possible to prevent severe forms of the disease and pre-eclampsia evolution to eclampsia, thus allowing the correct development and maturation of the fetus without risking the mother’s health and well-being.

## 4. Pathophysiology

Although it is a well-studied disease, the pathophysiology of pre-eclampsia remains uncertain. Several key features are thought to have a role in the development of pre-eclampsia, which is mainly considered as a vascular disorder. The most probable causes for this disease are a failure of trophoblast invasion leading to a failed transformation of the uterine spiral arteries, and an incorrect deep placentation [33]. Trophoblasts are the first cells that differentiate from the fertilized egg, they form the outer membrane of the placenta, and are responsible for the nutrients and oxygen exchange between the mother and the fetus [13,34]. Also, decidual natural killer (NK) cells can regulate trophoblast invasion and vascular growth, two essential processes in placental development [35]. An abnormal expression of NK cell surface antigens and a failure in the regulation of NK cell cytotoxicity and cytokines or angiogenic factors may be some of the causes of pre-eclampsia [36], resulting in a high-flow and high-pressure state [13,37,38]. Consequently, there is a high risk for ischemia-reperfusion injury of the placenta because of the vasoconstriction of the maternal arteries, which will lead to the formation of reactive oxygen radicals and further endothelial dysfunction [13,38,39]. Thus, pre-eclampsia can be related with the excessive release of some mediators by the injured endothelial cells.

The excessive soluble fms-like tyrosine kinase (sFlt)-1 or endoglin and the reduced free placental growth factor (PlGF) constitute another hypothesis for the pathogenesis of preeclampsia, namely, the angiogenic imbalance [34]. When sFlt-1 levels, which is a variant for PlGF and VEGF, are increased there is an inactivation or decrease of PlGF and VEGF concentration, resulting in endothelial dysfunction [34]. In the case of endoglin, which is a surface coreceptor for the transforming growth factor β (TGFβ) family, soluble endoglin (sEng) binds to endothelial receptors and inhibits several TGFβ isoforms, resulting in a decreased endothelial nitric oxide (NO)-dependent vasodilatation [40]. Vascular endothelial cells collected from pre-eclamptic women or exposed to serum from pre-eclamptic pregnancies produce less NO than endothelial cells from normal pregnancies [41,42,43]. Akar et al. demonstrated that agonist-stimulated NO production is reduced in isolated umbilical arteries [43,44]. Other studies also reported a decrease in agonist-stimulated NO production in umbilical and hand vein endothelial cells derived from pre-eclamptic pregnancies, concluding that the production of NO is compromised also in the maternal systemic arterial and venous vasculature, and not only in the maternal uterine and umbilical vasculature [42,45,46,47].

Prostacyclin (PGI_2_), another potent vasodilator, is decreased in pre-eclamptic women. This could be due to impaired endothelial Ca^2+^ signaling [42,43] and to the inhibition of PGI_2_ production by reactive oxygen species (ROS) [43,48]. It is still unclear the role of endothelium-derived hyperpolarizing factor (EDHF) in the vascular pathogenesis of pre-eclampsia, however, EDHF-mediated vasorelaxation is reduced in vessels from pre-eclamptic pregnancies [47,49,50].

A subset of women with pre-eclampsia have detectable autoantibodies against type-1 angiotensin II receptor (AT_1_) in the serum [51,52] which can activate AT_1_ in endothelial cells, vascular smooth muscle cells, and mesangial cells from the kidney glomerulus. AT_1_ autoantobodies have been shown to induce hypertension, proteinuria, glomerulus capillary endotheliosis, increased production of sVEGFR-1 (soluble Vascular Endothelial Growth Factor Receptor) and sEng, and to stimulate the synthesis of NADPH oxidase. These combined actions lead to oxidative stress, increased production of thrombin, fibrinolysis defect with fibrin deposition, and finally to an anti-angiogenic state [11,53,54]. Pre-eclampsia has also been associated with thrombocytopenia [55]. In fact, the role of platelet activation in pre-eclampsia has been evidenced through several features, including increased platelet size and reduced lifespan, increased maternal plasma levels of platelet factor 4 and β thromboglobulin, increased production of thromboxane B2 by platelets, and thrombi formation in the microcirculation of several target organs [11]. As it was mentioned before, PGl2, which has vasodilator actions and inhibits platelet aggregation, is decreased in women with pre-eclampsia, while thromboxane A2 is increased, leading to vasoconstriction and platelet aggregation. These will lead to vasospasm and platelet consumption, which are characteristic of pre-eclampsia [11]. Another important feature in pre-eclamptic women is the excessive thrombin generation. This may be due to different causes (endothelial cell dysfunction, platelet activation, chemotaxis of monocytes, proliferation of lymphocytes, neutrophil activation, or excessive generation of tissue factor in response to the activity of proinflammatory cytokines) ending in the deposition of fibrin in several organ systems [11]. Other factors have been implicated in the pathogenesis of pre-eclampsia, including genetic, environmental, and lifestyle factors. Genetic and environmental factors regulate several components that determine the susceptibility of a woman to the disease, like the predisposition to hypertensive disorders, autoimmune diseases, or diabetes (these factors predispose for pre-eclampsia) [11].

On the other hand, excessive weight (body mass index >35 Kg/m^2^) is an important risk factor for the disease, with a relative risk of 1.96 in a 95% confidence interval of 1.34–2.87 [16,56]. Several studies have focused on the measurement of different biomarkers for pre-eclampsia, including maternal body mass index, concluding that overweight and obesity are among the most important risk factors for pre-eclampsia, with an attributable risk percent of 64.9% when compared to women with a normal body weight [1,57,58]. However, the mechanisms by which obesity increases the incidence of pre-eclampsia are still to be discovered, nonetheless, several hypotheses have arisen. It was proposed that maternal obesity may reduce cytotrophoblast migration and uterine spiral arteries remodeling, leading to placental ischemia. Also, obesity promotes the increase of circulating antiangiogenic factors and proinflammatory pathways by placental ischemia, leading to the reduction of vascular NO levels and the increase of peripheral resistance, which may lead to the development of pre-eclampsia. Obesity is not by itself the promotor of pre-eclampsia, but other metabolic abnormalities are mandatory for obesity to increase the risk pre-eclampsia [59].

**Figure 1:**
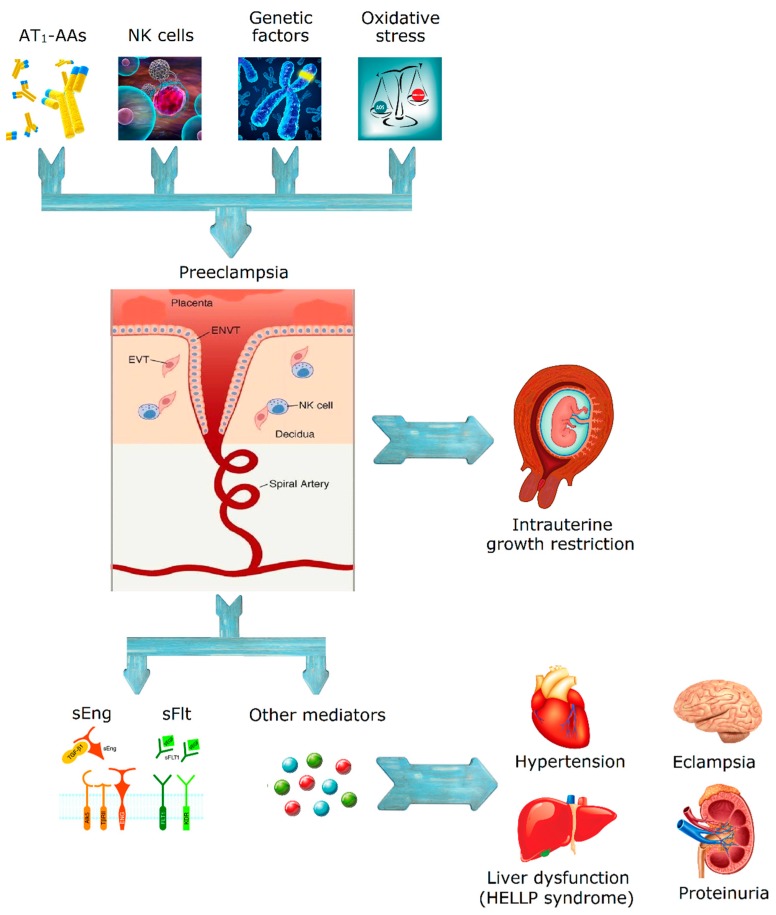
Proposed mechanism for pre-eclampsia and eclampsia.

Figure 1 summarizes the pathophysiology of the disease.

## 5. Pharmacological Therapy

For the prevention of pre-eclampsia, the only effective therapy that is currently known is low-dose aspirin. Some international guidelines, including those from the World Health Organization (WHO), have reported that, from 12 weeks of gestation until delivery, a dose of 75–100 mg of aspirin should be prescribed [56,60]. However, some studies demonstrated the benefits of this therapy only in women at high risk for the disease, in whom aspirin reduces the risk of preterm pre-eclampsia and the incidence of severe pre-eclampsia [61,62]. More recently, Tong et al. concluded that the aspirin dose should be greater than 100 mg and that, according to a study performed by Meher and collaborators, starting the aspirin after 16 weeks gestation is still beneficial to prevent pre-eclampsia [63,64].

One of the guidelines used in a Portuguese hospital also suggests the intake of aspirin (100 mg) by pregnant women with more than one risk factor [12]. Other preventive measures, including magnesium supplementation, fish oil supplementation, and vitamins C, D, and E supplements, have been proposed but failed to demonstrate a real benefit and receive consensus within the scientific community [65]. Calcium supplementation is related to a reduction in the risk of pre-eclampsia and in preterm birth [66]. It is most effective in populations where dietary calcium ingestion is low (<600 mg/day, which can occur in some low—and middle-income countries)—in these cases, WHO recommends a daily supplement of 2 g of calcium per day [66,67]. Regarding lifestyle interventions, several studies found no benefits in sodium restriction, diet interventions, and regular physical exercise [62,68]. The correct diagnosis and classification of the disease is essential, since the pharmacological therapy for the mild and severe forms of pre-eclampsia are distinct. The management of mild pre-eclampsia is intended to prevent the evolution to severe pre-eclampsia, to establish the timing of delivery, and to evaluate fetal lung development. In the case of severe pre-eclampsia, the objectives are the prevention of eclampsia (seizures), a rigorous control of blood pressure, and the planning of delivery. The most recent studies failed to prove the benefits of an antihypertensive therapy in pregnant women with mild pre-eclampsia in which the blood pressure is between 140/90 mmHg–150/100 mmHg: in these cases, medical surveillance is the only recommended measure [10]. Most guidelines, including some used in Portugal, follow this advice, suggesting that an antihypertensive therapy should be initiated only if SBP > 150–160 mmHg or if DBP > 100–110 mmHg [12,65,69,70].

It should be noted that angiotensin-converting enzyme (ACE) inhibitors and angiotensin receptor antagonists (ARA) should be avoided during pregnancy because of their teratogenic effects [9,12]. Also, it is important to avoid sublingual drug formulations, since they induce a rapid antihypertensive effect and can cause hypoperfusion of maternal target organs and potentially impair uteroplacentary circulation [65].

## 6. Mild Pre-Eclampsia

First, it is important to differentiate first-line and second-line therapies. The first-line therapy is the one accepted as the best treatment for the disease. This therapy can also be called induction therapy, primary therapy, and primary treatment. The second-line therapy is the treatment that is given when the primary treatment does not work or stops working. For this disease, oral alpha-methyldopa, 250 mg (2–3 tablets/day) or oral nifedipine, 30–60 mg in slow-release forms (once daily) can be considered as first-line treatment. Nifedipine is a calcium channel blocker described as a safe, effective, and nonteratogenic drug [7,71]. Alpha-methyldopa is an α-adrenergic receptor agonist which is also an effective and safe drug in pregnancy, but the fact that it needs to be taken more than once daily is a disadvantage with respect to nifedipine. In Portugal, alpha-methyldopa is also used as a valid and safe alternative to the calcium channel blockers like nifedipine, being used as second-line therapy for mild pre-eclampsia [12]. The NICE (*National Institute for Health and Care Excellence*) and NHS (*National Health Services*) guidelines recommend oral labetalol for mild pre-eclampsia, since this drug is the only antihypertensive drug approved in United Kingdom for pregnancy [65]. However, other consulted guidelines recommended intravenous labetalol only for the severe form of the disease. Table 3 states a proposed pharmacotherapy for mild pre-eclampsia.

## 7. Severe Pre-Eclampsia

Because of the elevated risks that this form of the disease implies for the pregnant woman, it is recommended immediate hospital admission and continuous monitoring. The antihypertensive therapy should be started promptly, and the clinicians should check for signs of imminent eclampsia (if needed, they should start a prophylactic anticonvulsive therapy) [56]. The recommended first-line therapy, which is agreed by the several national and international guidelines analyzed, is intravenous labetalol [12,65,70]. The infusion should start with a bolus of 20 mg in 2 min, followed by doses between 20–80 mg every 10 min (maximum cumulative dose: 300 mg) until the blood pressure is <150/100 mmHg. The normal maintenance dose is 6–8 mL/h. The objective is to maintain the blood pressure under the referred values [65]. Labetalol is an α1- and β-adrenergic antagonist, safe to use during pregnancy in situations of severe hypertension. This drug should not be used if the patient has asthma; alternatively, oral nifedipine, 10–20 mg in immediate-release forms, can be used. Intravenous hydralazine can also be used if the pregnant woman is refractory to either labetalol or nifedipine [12]. In Table 4, the proposed pharmacotherapy for severe pre-eclampsia is reported.

## 8. Eclampsia

The anticonvulsive therapy is the most important therapy for eclampsia (Table 5). The recommended drug to use is intravenous magnesium sulfate. The infusion should start with a bolus of 4–6 g in 20 min, followed by a maintenance dose of 2–3 g (rate of 50–75 mL/h of 50 mg/mL in a physiologic solution or glucose solution). The therapy must be maintained for 24 h after the last convulsive state, or post-partum [12]. During the administration of this drug, it is important to control systemic magnesium levels to avoid any problems related to hypermagnesemia (in extreme cases, this can cause muscle paralysis and cardiorespiratory arrest), therefore, clinicians must constantly monitor the respiratory frequency, diuresis, and patellar reflexes [9]. Although not universally accepted, intravenous diazepam can be used as an alternative. This drug is related to greater fetal and maternal mortality and should only be used if the pregnant woman is refractory to magnesium sulfate [60]. In Portugal, several hospitals follow this treatment with diazepam only when magnesium sulfate is contraindicated [12,24,69,70].

It should be noted that, besides the anticonvulsive therapy, an antihypertensive therapy similar to the one recommended for severe pre-eclampsia is mandatory.

## 9. Corticosteroids

The use of corticosteroids has great importance in the successful outcome of pregnancy, since it helps the correct development of fetal lungs and is neuroprotective for preterm fetuses [72]. This therapy is especially useful and important in premature newborns, since it reduces the respiratory discomfort and insufficiency in the newborn and improves the fetal outcome [56,70]. Corticotherapy is therefore recommended to a pregnant woman between 24 and 36 weeks of gestation, for whom delivery is probable or planned in the next seven days (maximum) (see Table 6) [56,70]. The corticosteroids most commonly used are intramuscular (IM) betamethasone and intravenous (IV) dexamethasone. These two drugs have very similar security and efficiency indexes [56,70].

## 10. Conclusions

The different guidelines available for the management of pre-eclampsia and eclampsia are not completely consensual in their content. The pharmacotherapy presented in this review is based on the recommendations from various guidelines for the disease, Portuguese and International. At present, the clinician’s experience and the patient’s symptoms and response to treatment are still the most important factors that determine the drug prescription.

Pre-eclampsia is still a serious threat, mainly in underdeveloped countries where its incidence and mortality rates are higher. In these countries, there is an urgent need in health policies to promote the proper care of women who suffer from this disease and to inform the populations about the alert signs and symptoms, and the risks of pre-eclampsia. In developed countries, the incidence of the disease has increased in the past years, but the negative outcomes for the mother and the fetus have decreased, as a result of the continuous improvement in hospital care and follow-up.

Apart from low-dose aspirin, there is still no effective preventive measure for all forms of pre-eclampsia, and the pharmacological management of the disease is the most important factor for the patient’s and the fetus’s well-being. Slow-release nifedipine is the most recommended drug for mild pre-eclampsia, alongside with alpha-methyldopa. For the severe form of the disease, labetalol is the recommended drug, being nifedipine and hydralazine the alternative drugs. For the prevention of seizures from eclampsia, magnesium sulfate is the drug of choice, and, in this case, although there is no established standard of care at this time, it is possible to use diazepam as an alternative. The administration of corticosteroids for fetal lung maturation has proven advantages in the fetal outcome and is recommended in pregnant women that are predicted to have a preterm delivery.

The importance of prescribing the correct therapy in pre-eclampsia and eclampsia is vital for mother and fetal outcomes, and all the hospital’s professional healthcare team (nurses, clinicians, pharmacists) have the responsibility to promote the correct use of the recommended drugs. Thus, we can conclude that, although there is no national guideline that allows a standardized and uniform treatment in all Portuguese hospitals, the guidelines developed and followed by these same hospitals go according to some international guidelines. However, there are still many discrepancies, as has been mentioned, and it would be worth adding a guideline whereby the professional healthcare team could be guided for a better health and prognosis of the patients.

## Figures and Tables

**Table 1:** Signs and symptoms of pre-eclampsia per organ system.

Systems	Signs/Symptoms
**Central Nervous system**	Headaches
	Visual disturbances
	Seizures (eclampsia)
**Renal system**	Proteinuria
	Oliguria
	Abnormal kidney tests
	Hypertension
**Vascular system**	Severe hypertension
**Cardiorespiratory system**	Chest pain
	Dyspnea
	Low oxygen saturation
	Pulmonary edema
**Hepatic system**	Abnormal liver function
	Epigastric pain
	Nausea
**Hematologic system**	Hemorrhage
	Coagulation impairment
	Intravascular disseminated coagulation
	Shock

**Table 2:** Summary of risk factors for pre-eclampsia.

Risk Factors for Pre-Eclampsia	Mean Relative Risk (95% Confidence Interval)	References
Antiphospholipid syndrome	9.72 (4.34–21.75)	[16]
Relative risk of preeclampsia	7.19 (5.85–8.83)	
Previous pre-eclampsia	7.19 (5.85–8.83)	
Diabetes mellitus (type I or II)	3.56 (2.54–4.99)	
Multiple pregnancy	2.93 (2.04–4.21)	
First pregnancy	2.91 (1.28–6.61)	
Familiar history of pre-eclampsia	2.90 (1.70–4.93)	
BMI ≥ 35 Kg/m^2^	2.47 (1.66–3.67)	
Maternal age <20 or >40 years old	1.96 (1.34–2.87)	
Chronic hypertension	1.38 (1.01–1.87)	
Chronic autoimmune disease	6.9 (1.1–42.3)	[19]
Venous thromboembolism (VTE)	2.2 (1.3–3.7)	[20]
Intergestational interval ≥10 years	Similar to multiple pregnancy	[21]
Chronic kidney disease	1.70 (1.30–2.23) *	[22]

* Values for odd ratio.

**Table 3:** Proposed pharmacotherapy for mild pre-eclampsia.

Mild Pre-Eclampsia		
Blood Pressure <150/100 mmHg	Blood Pressure ≥150/100 and <160/110 mmHg	
Expectant management. The pregnant woman should maintain: Rigorous control of blood pressure Bed rest Evaluate the necessity for hospital admission	**First line**	**Second line**
	**Nifedipine** per os, slow-release forms, 30–60 mg once a day (breakfast), max 120 mg/day	**Methyldopa** per os, 250–500 mg, 2–3 times per day (max 2–3 g/day) **Atenolol** per os, 50–100 mg/day

**Table 4:** Proposed pharmacotherapy for severe pre-eclampsia.

Severe Pre-Eclampsia		
First Line	Second Line	
Labetalol	Nifedipine	Hydralazine
Initiate bolus 20 mg IV (2 min) Repeat doses of 20–80 mg every 10 min (max cumulative dose: 300 mg) Maintenance dose: 6–8 mL/h (adjust between 2–12 mL/h according to patient’s evolution) from a concentration of 1 mg/mL	10–20 mg, immediate-release forms (never use sublingual administration)	Bolus 5 mg IV (2 min) Repeat doses every 20 min, until 20 mg total Maintenance dose: 2 mg/h

**Table 5:** Proposed pharmacotherapy for eclampsia prophylaxis.

Eclampsia		
Magnesium Sulphate		
Loading Dose	Maintenance Dose	“Booster” Dose (If Necessary)
4–6 g IV, slow infusion (20 min) 2–3 of 10 mL ampoules (20 mg/mL) in 100 mL of physiologic solution Perfusion at 200–300 mL/h	2–3 g IV 8 of 10 mL ampoules (50 mg/mL) in 1000 mL of physiologic solution or glucose solution Perfusion at 50–75 mL/h, maintain for 24 h after birth of after last seizure	2 g IV, slow infusion (10 min) 1 of 10 mL ampoule (20 mg/mL) if recurrent seizures
**If magnesium sulphate is contraindicated or if the patient is refractory to this treatment:** Diazepam, 5 mg IV (5 min), repeat until max dose (20 mg).		

**Table 6:** Proposed pharmacotherapy for fetal lung maturation.

Corticosteroids for Fetal Lung Maturation	
Corticotherapy should only be recommended if: Gestational age between 24 and 36 weeks Birth is planned or likely to happen in 7 days (limit)	
**Betamethasone**	**Dexamethasone**
12 g IM, 2 doses with a 24 h interval.	10 mg IV, 2 doses with a 24 h interval.

## References

[1] Gongora M.C., Wenger N.K. (2015). Cardiovascular complications of pregnancy. Int. J. Mol. Sci..

[2] Flack J.M., Peters R., Mehra V.C., Nasser S.A. (2002). Hypertension in special populations. Cardiol. Clin..

[3] Mustafa R., Ahmed S., Gupta A., Venuto R.C. (2012). A comprehensive review of hypertension in pregnancy. J. Pregnancy.

[4] Ahmad A.S., Samuelsen S.O. (2012). Hypertensive disorders in pregnancy and fetal death at different gestational lengths: A population study of 2 121 371 pregnancies. BJOG.

[5] Lindheimer M.D., Taler S.J., Cunningham F.G. (2010). Hypertension in pregnancy. J. Am. Soc. Hypertens..

[6] Say L., Chou D., Gemmill A., Tuncalp O., Moller A.B., Daniels J., Gulmezoglu A.M., Temmerman M., Alkema L. (2014). Global causes of maternal death: A who systematic analysis. Lancet Glob. Health.

[7] Folic M., Folic N., Varjacic M., Jakovljevic M., Jankovic S. (2008). Antihypertensive drug therapy for hypertensive disorders in pregnancy. Acta Med. Median..

[8] Kuklina E.V., Ayala C., Callaghan W.M. (2009). Hypertensive disorders and severe obstetric morbidity in the united states. Obstet. Gynecol..

[9] Moussa H.N., Arian S.E., Sibai B.M. (2014). Management of hypertensive disorders in pregnancy. Womens Health.

[10] Siqueira F., Moura T.R., Silva S.S., Peraçoli J.C. (2011). Medicamentos anti-hipertensivos na gestação e puerpério. Complementos Ciências Saúde.

[11] Chaiworapongsa T., Chaemsaithong P., Yeo L., Romero R. (2014). Pre-eclampsia part 1: Current understanding of its pathophysiology. Nat. Rev. Nephrol..

[12] Silva V., Palmira J., Martins N.N., Veríssimo R. (2014). Distúrbios hipertensivos. CHTV, EPE—Hospital são Teotónio, Viseu. Departamento de Obstetrícia e Ginecologia: Normas de Orientação Clínica.

[13] Dhariwal N.K., Lynde G.C. (2016). Update in the management of patients with preeclampsia. Anesthesiol. Clin..

[14] ACOG Committee on Obstetric Practice (2002). ACOG practice bulletin. Diagnosis and management of preeclampsia and eclampsia. Number 33, January 2002. American college of obstetricians and gynecologists. Int. J. Gynaecol. Obstet..

[15] Grand’Maison S., Pilote L., Okano M., Landry T., Dayan N. (2016). Markers of vascular dysfunction after hypertensive disorders of pregnancy: A systematic review and meta-analysis. Hypertension.

[16] English F.A., Kenny L.C., McCarthy F.P. (2015). Risk factors and effective management of preeclampsia. Integr. Blood Press Control.

[17] Xiong X., Wang F.L., Davidge S.T., Demianczuk N.N., Mayes D.C., Olson D.M., Saunders L.D. (2000). Maternal smoking and preeclampsia. J. Reprod. Med..

[18] Zdravkovic T., Genbacev O., McMaster M.T., Fisher S.J. (2005). The adverse effects of maternal smoking on the human placenta: A review. Placenta.

[19] Stamilio D.M., Sehdev H.M., Morgan M.A., Propert K., Macones G.A. (2000). Can antenatal clinical and biochemical markers predict the development of severe preeclampsia?. Am. J. Obstet. Gynecol..

[20] Bellamy L., Casas J.P., Hingorani A.D., Williams D.J. (2007). Pre-eclampsia and risk of cardiovascular disease and cancer in later life: Systematic review and meta-analysis. BMJ.

[21] Skjaerven R., Wilcox A.J., Lie R.T. (2002). The interval between pregnancies and the risk of preeclampsia. N. Engl. J. Med..

[22] Ayansina D., Black C., Hall S.J., Marks A., Millar C., Prescott G.J., Wilde K., Bhattacharya S. (2016). Long term effects of gestational hypertension and pre-eclampsia on kidney function: Record linkage study. Pregnancy Hypertens..

[23] Povoa A.M., Costa F., Rodrigues T., Patricio B., Cardoso F. (2008). Prevalence of hypertension during pregnancy in portugal. Hypertens. Pregnancy.

[24] Campos D.A., Silva I.S., Costa F.J., LIDEL (2011). Eclâmpsia. Emergências Obstétricas.

[25] Société française d’anesthésie et de réanimation (Sfar), Collège national des gynécologues et obstétriciens français (CNGOF), Société française de médecine périnatale (SFMP), Société française de néonatalogie (SFNN) (2009). [multidisciplinary management of severe pre-eclampsia (PE). Experts’ guidelines 2008. Societe francaise d’anesthesie et de reanimation. College national des gynecologues et obstetriciens francais. Societe francaise de medecine perinatale. Societe francaise de neonatalogie]. Ann. Fr. Anesth. Reanim..

[26] Stekkinger E., Zandstra M., Peeters L.L., Spaanderman M.E. (2009). Early-onset preeclampsia and the prevalence of postpartum metabolic syndrome. Obstet. Gynecol..

[27] Ray J.G., Vermeulen M.J., Schull M.J., Redelmeier D.A. (2005). Cardiovascular health after maternal placental syndromes (champs): Population-based retrospective cohort study. Lancet.

[28] Enkhmaa D., Wall D., Mehta P.K., Stuart J.J., Rich-Edwards J.W., Merz C.N., Shufelt C. (2016). Preeclampsia and vascular function: A window to future cardiovascular disease risk. J. Womens Health.

[29] Lin Y.S., Tang C.H., Yang C.Y., Wu L.S., Hung S.T., Hwa H.L., Chu P.H. (2011). Effect of pre-eclampsia-eclampsia on major cardiovascular events among peripartum women in taiwan. Am. J. Cardiol..

[30] Kestenbaum B., Seliger S.L., Easterling T.R., Gillen D.L., Critchlow C.W., Stehman-Breen C.O., Schwartz S.M. (2003). Cardiovascular and thromboembolic events following hypertensive pregnancy. Am. J. Kidney Dis..

[31] Kajantie E., Eriksson J.G., Osmond C., Thornburg K., Barker D.J. (2009). Pre-eclampsia is associated with increased risk of stroke in the adult offspring: The helsinki birth cohort study. Stroke.

[32] Davis E.F., Lazdam M., Lewandowski A.J., Worton S.A., Kelly B., Kenworthy Y., Adwani S., Wilkinson A.R., McCormick K., Sargent I. (2012). Cardiovascular risk factors in children and young adults born to preeclamptic pregnancies: A systematic review. Pediatrics.

[33] Fisher S.J. (2015). Why is placentation abnormal in preeclampsia?. Am. J. Obstet. Gynecol..

[34] Gathiram P., Moodley J. (2016). Pre-eclampsia: Its pathogenesis and pathophysiolgy. Cardiovasc. J. Afr..

[35] Hanna J., Goldman-Wohl D., Hamani Y., Avraham I., Greenfield C., Natanson-Yaron S., Prus D., Cohen-Daniel L., Arnon T.I., Manaster I. (2006). Decidual nk cells regulate key developmental processes at the human fetal-maternal interface. Nat. Med..

[36] Fukui A., Yokota M., Funamizu A., Nakamua R., Fukuhara R., Yamada K., Kimura H., Fukuyama A., Kamoi M., Tanaka K. (2012). Changes of nk cells in preeclampsia. Am. J. Reprod. Immunol..

[37] Tessier D.R., Yockell-Lelievre J., Gruslin A. (2015). Uterine spiral artery remodeling: The role of uterine natural killer cells and extravillous trophoblasts in normal and high-risk human pregnancies. Am. J. Reprod. Immunol..

[38] Burton G.J., Woods A.W., Jauniaux E., Kingdom J.C. (2009). Rheological and physiological consequences of conversion of the maternal spiral arteries for uteroplacental blood flow during human pregnancy. Placenta.

[39] Hung T.H., Skepper J.N., Burton G.J. (2001). In vitro ischemia-reperfusion injury in term human placenta as a model for oxidative stress in pathological pregnancies. Am. J. Pathol..

[40] Malik R., Kumar V. (2017). Hypertension in pregnancy. Adv. Exp. Med. Biol..

[41] Hayman R., Warren A., Brockelsby J., Johnson I., Baker P. (2000). Plasma from women with pre-eclampsia induces an in vitro alteration in the endothelium-dependent behaviour of myometrial resistance arteries. BJOG.

[42] Krupp J., Boeldt D.S., Yi F.X., Grummer M.A., Bankowski Anaya H.A., Shah D.M., Bird I.M. (2013). The loss of sustained Ca^2+^ signaling underlies suppressed endothelial nitric oxide production in preeclamptic pregnancies: Implications for new therapy. Am. J. Physiol. Heart Circ. Physiol..

[43] Goulopoulou S. (2017). Maternal vascular physiology in preeclampsia. Hypertension.

[44] Akar F., Ark M., Uydes B.S., Soysal M.E., Saracoglu F., Abacioglu N., Van de Voorde J., Kanzik I. (1994). Nitric oxide production by human umbilical vessels in severe pre-eclampsia. J. Hypertens..

[45] Steinert J.R., Wyatt A.W., Poston L., Jacob R., Mann G.E. (2002). Preeclampsia is associated with altered Ca^2+^ regulation and no production in human fetal venous endothelial cells. FASEB J..

[46] Mahdy Z., Otun H.A., Dunlop W., Gillespie J.I. (1998). The responsiveness of isolated human hand vein endothelial cells in normal pregnancy and in pre-eclampsia. J. Physiol..

[47] Boeldt D.S., Bird I.M. (2017). Vascular adaptation in pregnancy and endothelial dysfunction in preeclampsia. J. Endocrinol..

[48] Davidge S.T., Everson W.V., Parisi V.M., McLaughlin M.K. (1993). Pregnancy and lipid peroxide-induced alterations of eicosanoid-metabolizing enzymes in the aorta of the rat. Am. J. Obstet. Gynecol..

[49] Luksha L., Agewall S., Kublickiene K. (2009). Endothelium-derived hyperpolarizing factor in vascular physiology and cardiovascular disease. Atherosclerosis.

[50] Luksha L., Nisell H., Luksha N., Kublickas M., Hultenby K., Kublickiene K. (2008). Endothelium-derived hyperpolarizing factor in preeclampsia: Heterogeneous contribution, mechanisms, and morphological prerequisites. Am. J. Physiol. Regul. Integr. Comp. Physiol..

[51] Wallukat G., Homuth V., Fischer T., Lindschau C., Horstkamp B., Jupner A., Baur E., Nissen E., Vetter K., Neichel D. (1999). Patients with preeclampsia develop agonistic autoantibodies against the angiotensin at1 receptor. J. Clin. Investig..

[52] Harmon A.C., Cornelius D.C., Amaral L.M., Faulkner J.L., Cunningham M.W., Wallace K., LaMarca B. (2016). The role of inflammation in the pathology of preeclampsia. Clin. Sci..

[53] Parrish M.R., Murphy S.R., Rutland S., Wallace K., Wenzel K., Wallukat G., Keiser S., Ray L.F., Dechend R., Martin J.N. (2010). The effect of immune factors, tumor necrosis factor-alpha, and agonistic autoantibodies to the angiotensin ii type i receptor on soluble fms-like tyrosine-1 and soluble endoglin production in response to hypertension during pregnancy. Am. J. Hypertens..

[54] Xia Y., Kellems R.E. (2013). Angiotensin receptor agonistic autoantibodies and hypertension: Preeclampsia and beyond. Circ. Res..

[55] Yan M., Malinowski A.K., Shehata N. (2016). Thrombocytopenic syndromes in pregnancy. Obstet. Med..

[56] National Institute for Health and Care Exellence (NICE) (2015). Severe Hypertension, Severe Pre-Eclampsia and Eclampsia in Critical Care—Nice Clinical Guideline.

[57] Kenny L.C., Black M.A., Poston L., Taylor R., Myers J.E., Baker P.N., McCowan L.M., Simpson N.A., Dekker G.A., Roberts C.T. (2014). Early pregnancy prediction of preeclampsia in nulliparous women, combining clinical risk and biomarkers: The screening for pregnancy endpoints (scope) international cohort study. Hypertension.

[58] Pare E., Parry S., McElrath T.F., Pucci D., Newton A., Lim K.H. (2014). Clinical risk factors for preeclampsia in the 21st century. Obstet. Gynecol..

[59] Spradley F.T. (2016). Metabolic abnormalities and obesity’s impact on the risk for developing preeclampsia. Am. J. Physiol. Regul. Integr. Comp. Physiol..

[60] WHO, World Health Organization (2011). Who Recommendations for Prevention and Treatment of Pre-Eclampsia and Eclampsia.

[61] Roberge S., Villa P., Nicolaides K., Giguere Y., Vainio M., Bakthi A., Ebrashy A., Bujold E. (2012). Early administration of low-dose aspirin for the prevention of preterm and term preeclampsia: A systematic review and meta-analysis. Fetal Diagn. Ther..

[62] Mol B.W., Roberts C.T., Thangaratinam S., Magee L.A., de Groot C.J., Hofmeyr G.J. (2015). Pre-eclampsia. Lancet.

[63] Tong S., Mol B.W., Walker S.P. (2017). Preventing preeclampsia with aspirin: Does dose or timing matter?. Am. J. Obstet. Gynecol..

[64] Meher S., Duley L., Hunter K., Askie L. (2017). Antiplatelet therapy before or after 16 weeks’ gestation for preventing preeclampsia: An individual participant data meta-analysis. Am. J. Obstet. Gynecol..

[65] National Collaborating Centre for Women’s and Children’s Health (2010). Hypertension in Pregnancy: The Management of Hypertensive Disorders during Pregnancy—Nice Clinical Guideline.

[66] Hofmeyr G.J., Lawrie T.A., Atallah A.N., Duley L., Torloni M.R. (2014). Calcium supplementation during pregnancy for preventing hypertensive disorders and related problems. Cochrane Database Syst. Rev..

[67] WHO, World Health Organization (2013). Guideline: Calcium Supplementation in Pregnant Women.

[68] Inversetti A., Smid M., Candiani M., Ferrari M., Galbiati S. (2014). Predictive biomarkers of pre-eclampsia and effectiveness of preventative interventions for the disease. Expert Opin. Biol. Ther..

[69] Montenegro N., Campos D.A., Rodrigues T., Ramalho C., Silva J.L., Machado A.P., LIDEL (2014). Pré-eclâmpsia: Vigilância e tratamento. Protocolos de Medicina Materno-Fetal.

[70] Júlio C., Francisco C., Dias E., Campos A., LIDEL (2011). Pré-eclâmpsia. Protocolos de Atuação da Maternidade dr. Alfredo da Costa.

[71] Podymow T., August P. (2011). Antihypertensive drugs in pregnancy. Semin. Nephrol..

[72] Bouet P.E., Brun S., Madar H., Baisson A.L., Courtay V., Gascoin-Lachambre G., Lasocki S., Sentilhes L. (2015). Implementation of an antenatal magnesium sulfate protocol for fetal neuroprotection in preterm infants. Sci. Rep..

